# State-level data on TANF policy changes during the COVID-19 pandemic

**DOI:** 10.1186/s13104-023-06351-w

**Published:** 2023-06-05

**Authors:** Emily C. Dore, Paul R. Shafer, Melvin D. Livingston

**Affiliations:** 1 Department of Sociology, Emory University, 1555 Dickey Drive, Atlanta, GA 30322 USA; 2 Boston University School of Public Health, 715 Albany Street, Boston, MA 02118 USA; 3 Emory University, Rollins School of Public Health, 1518 Clifton Road, Atlanta, GA 30322 USA

**Keywords:** TANF, Temporary Assistance for Needy Families, Welfare, Work requirements, Sanctions, Time limits, Cash benefits, COVID-19

## Abstract

**Objective:**

COVID-19 mitigation measures prompted many states to revise the administration of their welfare programs. States adopted policies that varied across the U.S. to respond to the difficulties in fulfilling program requirements, as well as increased financial need. This dataset captures the changes made to Temporary Assistance for Needy Families (TANF) programs during the COVID-19 pandemic, from March 2020 through December 2020. The authors created this dataset as part of a larger study that examined the health effects of TANF policy changes during the COVID-19 pandemic.

**Data description:**

TANF is the main cash assistance program for low-income families in the U.S., but benefits are often conditional on work requirements and can be revoked if an individual is deemed noncompliant. Structural factors during the COVID-19 pandemic made meeting these criteria more difficult, so some states relaxed their rules and increased their benefits. This dataset captures 24 types of policies that state TANF programs enacted, which of the states enacted each of them, when the policies went into effect, and when applicable, when the policies ended. These data can be used to study the effects of TANF policy changes on various health and programmatic outcomes.

## Objective

Although funded by a federal block grant, TANF is a state-run cash assistance program. States have flexibility on how to run their TANF program, which creates high levels of variability between states in terms of generosity, administrative burden, and punishments for non-compliance [1]. As a block grant program, TANF funding does not change easily in the short-term, especially when there is no change on the federal level. However, during the COVID-19 pandemic, beneficiaries faced additional barriers to receiving assistance due to COVID-19 mitigation measures [2, 3]. For example, TANF assistance is commonly conditional on meeting work activity requirements [4]. Meeting these requirements became more difficult during the COVID-19 pandemic as stay at home orders proliferated, businesses closed, and individuals attempted to keep themselves and their families safe from the virus. As a result, some states modified their TANF practices to avoid beneficiaries losing benefits during a time of great need [2]. This dataset captures the varying policies and timelines of implementation by state. Specifically, the dataset contains information on whether each of the 50 states implemented changes to TANF policy between March 2020 and December 2020.

Researchers can match this dataset with other data on economic and health outcomes to understand how variations in TANF policy can affect economic and health outcomes. Policymakers can use these data to inform the adoption or modification of policies to best serve their constituents.

Dore et al. [5] analyzed this dataset to understand the effect of TANF policy changes during COVID-19 on stress-related health outcomes. They found that overall, more supportive policies led to better stress-related physical, mental, and behavioral health outcomes.

## Data description

The dataset is a table that contains the 24 TANF policy categories that represent changes to TANF state policy during the COVID-19 pandemic, the state names and their FIPS codes, and if the states enacted the policies, the start date, and when applicable, the end date. If the cell is empty in the dataset, the state did not implement that particular policy. Examples of policy categories include waiving in person interviews, pausing or lifting existing sanctions, automatically extending or recertifying benefits, allowing participants who reached time limits during the pandemic to continue receiving benefits, and providing additional temporary payments to families. Since the dataset describes the period of March 2020 through December 2020, we created two datasets to represent the ongoing nature of the pandemic. There is only one small difference between the two datasets. One dataset is ready for analysis and includes “December 2020” in the end date cells for policies that had not ended by December 2020. However, these same cells are labeled as “potentially ongoing as December 2020” in the second dataset to clarify the fluid nature of these policies for researchers who may want to extend the analysis beyond December 2020.

To compile this dataset, we first gathered the policy categories from the Center for American Progress website [2]. We then systematically confirmed the implementation of the policy for each of the states listed on that website. The author of the original article, Justin Schweitzer, provided us with a comprehensive list of his sources. We confirmed first using these sources for almost all of the policies. We were unable to confirm some of the policies because his sources came from personal emails or from websites that no longer existed, but we trusted their accuracy. Next, we moved on to finding the start and end dates, which the original article did not contain. We went back to the original sources to identify dates where possible and noted them in the table. When those sources did not contain dates (end dates were especially lacking), we checked other sources online. These sources were: governors’ executive orders, local news articles, legislation, state websites, and email correspondence with state TANF staff. Executive orders and state websites were particularly useful. If we did not find information on the state website (including the TANF website, the governor’s office, or legislative records) or through local news channels directly, we broadened our search using a combination of search terms. The search terms included: TANF, the state-specific TANF program name (e.g. Iowa’s TANF program is called “The Family Investment Program”), a descriptor of the policy (e.g. “work requirement”), COVID-19, coronavirus, pandemic, press release, executive order, legislature, governor, local news, etc. Due to lack of documentation, there were several instances when dates had to be inferred or assumed. The way we did this depended on the source of the information, and we describe our process in more detail in the methodology document in the data depository.

**Table 1:** Overview of data files and data sets

Label	Name of data file/data set	File types	Data repository and identifier
*Data set 1*	*TANF COVID-19*	*MS Excel file (.xlsx)*	Emory Dataverse 10.15139/S3/79WVWC [6]
*Data set 2*	*TANF COVID-19_ongoing*	*MS Excel file (.xlsx)*	Emory Dataverse 10.15139/S3/79WVWC [6]
*Data file 1*	*Dataset Methodology*	*MS Word file (.docx)*	Emory Dataverse 10.15139/S3/79WVWC [6]

## Limitations

Although we did our best to compile a thorough and accurate timeline and record of policy changes during the first several months of the pandemic, there are some limitations to the dataset. First, sometimes it was not possible to find confirmed start and end dates. In these cases, we made informed estimates using a standardized process based on available information. We include more information on this process in the Methodology document available through Emory Dataverse, but in general, we assumed dates based on dates of other policies implemented in that state, or dates of that same policy implemented in other states. Second, we did not look beyond the list of policies provided by the Center for American Progress for additional policies that may have been implemented. It is possible there were other changes to TANF policy that we missed, though we believe the policy dimensions captured to be the most salient. Lastly, our dataset ends in December 2020 to align with the Center for American Progress list of policies. This period is likely the period with the most changes due to the urgency surrounding the first few months of the pandemic, though we are aware of other policies that were implemented afterwards. For example, more states have since provided emergency cash assistance and the federal government has implemented new TANF policies. However, unemployment was at its highest in the US between March 2020-December 2020 [6] and while TANF caseloads increased beginning in March 2020, they returned to pre-pandemic levels by November 2020 [3]. Thus, we believe our dataset captures the most important time during which individuals needed the most help.

## Data Availability

The data described in this Data note are publicly available and can be freely and openly accessed on Emory Dataverse under 10.15139/S3/79WVWC. Please see Table 1 and references [5, 6] for details and links to the data. This article is licensed under a Creative Commons Attribution 4.0 International License, which permits use, sharing, adaptation, distribution and reproduction in any medium or format, as long as you give appropriate credit to the original author(s) and the source, provide a link to the Creative Commons license, and indicate if changes were made. The images or other third party material in this article are included in the article’s Creative Commons license, unless indicated otherwise in a credit line to the material. If material is not included in the article’s Creative Commons license and your intended use is not permitted by statutory regulation or exceeds the permitted use, you will need to obtain permission directly from the copyright holder. To view a copy of this license, visit http://creativecommons.org/licenses/by/4.0/. The Creative Commons Public Domain Dedication waiver (http://creativecommons.org/publicdomain/zero/1.0/) applies to the data made available in this article, unless otherwise stated in a credit line to the data.

## Abbreviations

TANF: Temporary Assistance for Needy Families

## References

[1] Schott L, Pavetti L, Floyd I. How States Use Federal and State Funds Under the TANF Block Grant [Internet]., Washington DC. Center on Budget and Policy Priorities; 2015 Oct [cited 2021 Dec 13]. Available from: https://www.cbpp.org/research/family-income-support/how-states-use-federal-and-state-funds-under-the-tanf-block-grant.

[2] Schweitzer J. How States Can Use TANF To Immediately Help Struggling Residents [Internet]. The Center for American Progress; 2020 Dec [cited 2022 Apr 29]. Available from: https://www.americanprogress.org/article/states-can-use-tanf-immediately-help-struggling-residents/.

[3] Hembre E, Examining SNAP, Caseload Trends TANF. Responsiveness, and Policies during the COVID-19 Pandemic [Internet]. Rochester, NY: Social Science Research Network; 2021 Jul [cited 2022 Apr 28]. Report No.: 3693339. Available from: http://papers.ssrn.com/abstract=3693339.

[4] Shantz K, Dehry I, Knowles S, Minton S, Giannarelli L. Welfare Rules Databook: State TANF Policies as of July 2019 [Internet]. Washington, D.C.: The Urban Institute; 2019 Jul [cited 2021 Dec 2] p. 1–302. Report No.: 2020–141. Available from: https://www.acf.hhs.gov/opre/report/welfare-rules-databook-state-tanf-policies-july-2019.

[5] Dore EC, Livingston MD III, Shafer PR. Easing Cash Assistance Rules During COVID-19 Was Associated With Reduced Days of Poor Physical and Mental Health. Health Affairs; 2022 Nov; 41(11):1590–7. 10.1377/hlthaff.2022.00740 PMC13587817 36343321

[6] Dore EC, Shafer PR, Livingston MD. State-Level Data on TANF Policy Changes During the COVID-19 Pandemic [Internet]. Emory Dataverse; 2022. Available from: 10.15139/S3/79WVWC. 10.1186/s13104-023-06351-w PMC10241382 37277783

